# Tetracycline Resistance and Presence of Tetracycline Resistance Determinants *tet*(V) and *tap* in Rapidly Growing Mycobacteria from Agricultural Soils and Clinical Isolates

**DOI:** 10.1264/jsme2.ME12028

**Published:** 2012-05-17

**Authors:** Martina Kyselková, Alica Chron̂áková, Lucie Volná, Jan Nêmec, Vít Ulmann, Josef Scharfen, Dana Elhottová

**Affiliations:** 1 Biology Centre of the Academy of Sciences of the Czech Republic, Institute of Soil Biology, České Budějovice, Czech Republic; 2 Institute of Public Health, Ostrava, Czech Republic; 3 Institute of Clinical Microbiology, Charles University in Prague, Faculty of Medicine in Hradec Králové and University Hospital Faculty of Medicine in Hradec Králové, Trutnov, Czech Republic; 4 National Reference Laboratory for Pathogenic Actinomycetes, Dept. Medical Microbiology and Immunology, Regional Hospital Trutnov, Inc., Trutnov, Czech Republic

**Keywords:** efflux pump, rapidly growing *Mycobacterium*, tetracycline resistance, *tap*, *tet*(V)

## Abstract

Rapidly growing mycobacteria (RGM) inhabit soil and water but certain strains represent a health risk for human and animals. Both clinical and soil RGM may be under selection pressure for resistance to tetracycline (TET) antibiotics, since tetracyclines are administrated to humans and farm animals, and TET residues enter soil through manuring; however, resistance to TET and the presence of TET-resistance genes have been assessed only in clinical isolates. We were therefore interested in comparing soil and clinical RGM in terms of TET resistance and the presence of TET-resistance genes. We used 44 RGM from grasslands with different exposure to animal manure, and 38 clinical RGM from Czech hospitals. There was no difference between the clinical and soil isolates in TET resistance, with >50% resistant isolates in both groups. *otr*(A), *otr*(B), *tet*(K), *tet*(L) or *tet*(M) were not detected in any soil or clinical isolate. In contrast, most isolates harbored *tet*(V) and *tap*, both encoding mycobacterial efflux pumps, including species where these genes have never been evidenced before. The phylogeny of *tet*(V) correlated with isolates’ BOX-PCR profiles, suggesting that this gene evolved along with mycobacterial genomes as a part of the intrinsic resistome. In certain cases, *tet*(V) and/or *tap* were found in TET-sensitive isolates, or inversely, were not found in resistant strains. Concluding, intrinsic efflux pumps may be more important for TET resistance than horizontally transferred genes in both soil and clinical RGM. Their simple presence, however, does not attest to resistance, and therefore their diversity, function and expression merit further research.

Rapidly growing mycobacteria (RGM) are common inhabitants of soil and water. In past decades, they have been increasingly recognized as a cause of human and animal diseases (15, 25). These include respiratory infections, a spectrum of hard and soft tissue infections or bacteremia in immunocompromised patients, as well as infections related to injuries in healthy individuals (8, 29, 48). The natural and man-transformed environment is an important reservoir of RGM, from which transmission to humans occurs (50). Although the direct relationship between an environmental source and clinical disease is difficult to evidence, several RGM outbreaks have been associated with the exposure to soil and water (2, 16), including drinking water (49).

The prevention and treatment of infections due to RGM is not trivial because of their high resistance to disinfectants and antibiotics. One group of antibiotics used in the therapy of RGM infections is the tetracyclines (TET) such as doxycycline and minocycline (15). Mycobacteria in general have intrinsic resistance to many antibiotics ensured by the composition of their cell wall and the presence of several multidrug efflux pumps (33, 36). Tetracycline/multidrug efflux pumps Tet(V) and Tap may belong to this intrinsic resistome as they have been so far found only in certain RGM species (17, 22).

Acquired resistance is not often seen in *Mycobacterium*; however, Pang *et al.* (38) reported four genes conferring TET resistance in clinical RGM that have been most probably horizontally transferred from other bacteria. These included *otr*(A) and *otr*(B), self-protection genes from the oxytetracycline producer *Streptomyces rimosus* (9), and *tet*(K) and *tet*(L), low-G+C-content genes encoding tetracycline efflux pumps, which are typically found in *Firmicutes (Streptococcus*, *Staphylococcus*, *Enterococcus*) but also in some Gram-negative bacteria (40). More recently, *tet*(M), a widely distributed gene with a low G+C content encoding a ribosomal protection protein, was found in a human-associated *Mycobacterium* sp. (41).

Resistance to TET and the presence of TET-resistance genes in RGM have been studied in clinical isolates only; however, soil is an important reservoir of TET resistance genes, both indigenous (14) and introduced by manuring (10). In addition, tetracycline residues are detectable in manured soil (10), which may help select resistant strains. Soildwelling RGM may therefore represent an important pool of TET resistance genes.

The main objective of this study was to compare soil and clinical isolates of RGM (both from the Czech Republic) in terms of the resistance to tetracycline and presence of seven TET-resistance determinants. The genome relatedness of isolates was assessed with BOX-PCR as a high correlation exists between BOX-PCR fingerprints and DNA-DNA homology data (31, 39, 51). 16S rRNA gene sequencing was performed to identify the isolates. Resistance to TET was assessed with the agar disk diffusion method, and the presence of TET resistance genes that were previously described in clinical RGM, *i.e.*, *otr*(A), *otr*(B), *tet*(K), *tet*(L), *tet*(M), *tet*(V) and the multi-drug efflux pump-encoding gene *tap*, was checked with PCR and sequencing. The phylogeny of *tet*(V) was compared to the BOX-PCR profiles of isolates.

## Materials and Methods

### Soil isolates of rapidly-growing mycobacteria

Soil RGM were isolated from four sites of three farms located in South Bohemia, Czech Republic in 2007–2010 (Table S1). The distance between farms was up to 10 km. Farm 1 is a conventional farm engaged in intensive pig fattening, where animals (about 2,000) are commonly treated with antibiotics including chlortetracycline and doxycycline. At Farm 1, we sampled a permanent grassland, which had been periodically manured (2–3 times per year) with pig slurry for the previous 30 years (designated Site 1). At Farm 2, which is a small family farm in a neighborhood community, we sampled permanent grassland that had not been manured for the previous 20 years (Site 2). Samples were taken from Site 1 and Site 2 in June 2007 and 2009. Farm 3 has performed outdoor cattle husbandry since 1993 and is an organic farm without the application of antibiotics. Two sites (Site 3 and Site 4) were sampled at Farm 3 in May 2010. Site 3 is part of the pasture where cattle stay from October until May. It is highly impacted by the cattle, *i.e.*, the soil is highly enriched with excrement and vegetation cover is damaged (28). Site 4 is a pasture with low impact by the cattle and preserved vegetation. At each site, soil from a depth of about 10–30 cm (under the plant roots) was sampled with a sterile spade from three points 5–20 m apart. The soil from the three points was mixed and sieved. Soils were kept at 4°C during transport to the laboratory and prior to the accompanying physicochemical and microbial analyses (Table S1). RGM were isolated from soil using the NaOH/malachite green/cycloheximide decontamination method of Iivanainen (27) or the olive oil/SDS decontamination method (with 10 mg SDS per plate) of Yamamura and Harayama (53) or directly on Tryptic-Soy agar plates with 25 mg L^−1^ chlortetracycline (2 isolates from 2007) (Table S2).

### Clinical isolates of rapidly-growing mycobacteria

Clinical RGM were obtained from the National Reference Laboratory for Pathogenic Actinomycetes, Regional Hospital in Trutnov, and from the Institute of Public Health, Ostrava, Czech Republic. They were isolated in 2006–2011 from various samples such as abscess, urine, hemoculture, corneal ulcer and sputum from 18 hospitals in the Czech Republic (Table S3). Their role in the etiology of infection was confirmed in isolates from abscesses, hemocultures and corneal ulcers. Incidental isolates from sputa of patients screened for *Mycobacterium tuberculosis* without clinical and imaging correlates were usually colonizers, with the exception of *Mycobacterium chelonae* OS10 associated with a pulmonary disease in a 47-year-old patient.

### Susceptibility to tetracycline

Susceptibility to tetracycline was assessed with a disc diffusion test (26). Pure isolates were first grown on M2 (42) or Šula’s medium (43) with the addition of 1.5% agar, at 28°C for 5 to 7 d. A homogenous bacterial suspension was prepared by vortexing (Vortex-Genie2; Mo Bio Laboratories, Carlsbad, CA, USA) and ultrasonication (Ultrasonic Compact Cleaner UC 006DM1, Tesla, Czech Republic) of several colonies in 4 mL of sterile 0.9% NaCl. The turbidity of the suspension was adjusted with sterile 0.9% NaCl to match the McFarland standard 0.5 (densitometer DEN-1; Biosan, Latvia) (52). The suspension was spread onto Mueller-Hinton agar medium (Bio-Rad Laboratories, Hercules, CA, USA) supplied with TET disks (30 μg; Bio-Rad). Inhibition zone diameters were recorded after 5 d of incubation at 28°C. The ranked zone sizes of environmental and clinical strains were statistically compared with Wilcoxon rank sum test in R (http://www.r-project.org). Strains OS18, OS2/1, OS2/2 and OS2/4 did not grow on Mueller-Hinton agar and disc diffusion analysis was therefore performed on Šula’s medium. These strains were not included in the statistical analysis.

### BOX-PCR genomic DNA fingerprints

Prior to PCR amplification, cell lysates were prepared as follows. One bacteriological loop of mycobacterial biomass grown on an agar plate was resuspended in 100 μL ultra-pure water. The suspensions were then boiled three times (water bath, 100°C) for 5 min and frozen at −20°C for 1 h. The lysates were stored at −20°C and 1 μL of the lysates was used as a template for PCR.

DNA amplification followed the procedure of Lanoot *et al.* (31) using the BOXA1R primer (5′-CTACGGCAAGGCGACGCT GACG-3′) (51). PCR products (20 μL) were separated on 20×20 cm gels using 130 V, 400 mA for 240 min in 1×TBE buffer (Tris base 53 g, boric acid 27.5 g, 0.5 M EDTA 20 ml, pH 8.0). The gels were stained for 30 min in a 1×TBE bath supplemented with ethidium bromide (1 mg L^−1^). A photograph of the gel was stored as a TIFF file through a CCD coupled camera using Photo-Doc software (Vilber-Lourmat, Marne-la-Vallée, France). Gels were imported into the software package GelCompar II (Applied Maths, Sint-Martens-Latem, Belgium) and similarity matrices of densitometric curves of the gel tracks were calculated using the Pearson correlation coefficient followed by dendogram construction using the UPGMA algorithm. We used the limit of 70% similarity to define distinct BOX-PCR groups (12).

### Isolate identification

Clinical strains TR-1378, OS1, OS8, OS9, OS10, OS11, OS13, OS21, OS24, OS25, OS28, OS30, OS2/7 and OS2/8 were identified with the GenoType Mycobacterium CM (Common Mycobacteria) Test based on DNA Strip technology (Hain Lifescience, Nehren, Germany). The 16S rRNA gene of the clinical strain TR-1380 was sequenced by the commercial system MicroSeq 500 16S rDNA Bacterial Identification Kit (Applied Biosystems, Foster City, CA, USA). Both analyses were performed at the Institute of Public Health Ostrava (Czech Republic).

The remaining clinical isolates and at least one soil isolate from each BOX-PCR group were identified with 16S rRNA gene amplification using universal bacterial primers (21) pA (5′-AGAGTTTGATCCTGGCTCAG-3′) and pH (5′-AAGGAGGT GATCCAGCCGCA-3′), and sequencing. The total volume of PCR reactions was 50 μL. The final reaction mixtures contained (final concentrations) Expand Long Template PCR System Buffer #1 (Roche Applied Science, Mannheim, Germany; 1×), dNTPs (Fermentas, Thermo Fisher Scientific, Waltham, MA, USA; 0.3 mM each), primers (500 nM each) and Expand Long Template polymerase (Roche; 0.05 U μL^−1^). Mycobacterial lysate (1 μL, see above) served as a template. Thermal cycling was performed as follows: Initial denaturation at 94°C for 2 minutes; followed by 35 cycles of denaturation (94°C/15s), annealing (61°C/30s) and extension (68°C/45s; the duration of extension was prolonged to 90s after the first ten cycles); and final extension at 68°C for 7 min. Amplified 16S rRNA genes were cleaned-up with the GenElute PCR Clean-Up Kit (Sigma-Aldrich, St. Louis, MO, USA) and sequenced using the primers pA, pH and 519r (21, 30).

The obtained 16S rRNA gene sequences were edited by Bioedit 7.0.4.1 software (23) and assembled using SeqMAN (DNAStar, Madison, WI, USA) (45). The edited sequences were compared against the database of type strains *Ez-Taxon Database* (http://www.eztaxon.org) (11) to retrieve the most relative species.

### Detection of tetracycline resistance genes

The presence of tetracycline resistance genes *otr*(A), *otr*(B), *tet*(K), *tet*(L), *tet*(M), *tet*(V) and *tap* was assessed with PCR using gene-specific primers (Table 1). The total volume of PCR reactions was 25 μL and 1 μL of mycobacterial lysates (see above) or ~20–50 ng of chromozomal DNA from positive control strains or plasmid DNA were used as a template. The cycling conditions for each gene are shown in Table 1.

The genes *otr*(A), *otr*(B) and *tap* were amplified with the help of Qiagen Taq polymerase and Q-solution (Qiagen, Hilden, Germany). The final reaction mixtures contained (final concentrations) Qiagen Taq buffer (1×), Qiagen Q-solution (1×), dNTPs (Fermentas, Thermo Fisher Scientific; 0.2 mM each), primers [500 nM in the case of *otr*(A) and *tap*, and 100 nM in the case of *otr*(B)] and Qiagen Taq polymerase (0.05 U μL^−1^). The genes *tet*(K) and (L) (co-amplified), *tet*(V) and *tet*(M) were amplified using Dream Taq polymerase (Fermentas, Thermo Fisher Scientific) and the final reaction mixtures contained Dream Taq buffer [with (NH_4_)_2_SO_4_, without MgCl_2_; 1×], MgCl_2_ (1.5 mM), dNTPs (0.2 mM each), primers (500 nM each), dimethyl sulfoxide (Sigma-Aldrich; 5%), bovine serum albumin (Fermentas, 1.2 mg mL^−1^) and Dream Taq polymerase (0.05 U μL^−1^). Five microliters of PCR products were analyzed in 1–2% agarose gel (Top Vision agarose, Fermentas, Thermo Fisher Scientific) stained with ethidium bromide (1 mg L^−1^), 30 min, for the presence of bands of the expected size.

The specificity of primer pairs designed in this study, *i.e.*, tetKL-FW/tetKL-RV, and tetV-FW/tetV-RV was tested by PCR with other tetracycline efflux pump genes as a template (negative controls). The tetKL-FW/tetKL-RV were tested against plasmids containing genes *tet*(A), *tet*(B), *tet*(C), *tet*(D), *tet*(E), *tet*(G), *tet*(H), *tet*(J), *tet*(V), *tet*(Y), *tet*(Z) and agaist *Streptomyces rimosus* subsp. *rimosus* DSMZ 40260 chromosomal DNA containing *otr*(B). Primers tetV-FW/tetV-RV were tested against *tet*(A), *tet*(B), *tet*(C), *tet*(D), *tet*(E), *tet*(H), *tet*(J), *tet*(K), *tet*(L), *tet*(Y), *tet*(Z) and *otr*(B) (6, 7).

Amplified *tet*(V) or *tap* genes were cleaned-up with the GenElute PCR Clean-Up Kit (Sigma-Aldrich) and sequenced from both ends in the case of *tet*(V) and from the forward primer in the case of *tap*, as described above. The sequences were edited and assembled with Bioedit 7.0.4.1 software (23) and compared to the GenBank database (www.ncbi.nml.nih.gov) using blastn and blastp algorithms. Altogether, 46 PCR products of *tet*(V) and 14 PCR products of *tap* were sequenced.

### Phylogeny analyses of *tet*(V)

Phylogeny analyses were conducted in MEGA software version 4.0 (http://www.megasoftware.net/) (46). The partial sequences of *tet*(V) (311 nucleotides) were aligned together with the published sequence of *M. smegmatis tet*(V) (GenBank, gb|CP000480.1|: 5286339–5287598) and *M. vanbaalenii* H+ antiporter gene (Gen-Bank, CP000511.1). The neighbor-joining phylogenetic tree was constructed based on the nucleotide sequences using Kimura-2 parameter. The correlation between the distance matrices of *tet*(V) sequences of 43 isolates and corresponding BOX-PCR profiles was assessed with the Mantel test in R (http://www.r-project.org/), ADE-4 package (47), using 1,000 repetitions.

### Accession numbers

The *tet*(V) sequences were deposited in GenBank under accession numbers JF290326–JF290351 and JQ348076–JQ348095, and the *tap* sequences under accession numbers JF290352–JF290365. The 16S rRNA gene sequences are available in GenBank under accession numbers JF304573–JF304610 and JQ348096–JQ348111.

## Results

### Isolate identification and genome relatedness

Forty-four isolates from grasslands and 38 clinical isolates were included in this study (Table S2, S3 and S4). The environmental isolates were divided into 14 distinct groups and the clinical isolates into 28 distinct groups according to their BOX-PCR profile similarity, using the Pearson correlation coefficient threshold of 0.7 (Table 2, 3 and Fig. S1). Clinical and environmental strains always belonged to separate BOX-PCR groups.

A minimum of one isolate from each environmental BOX-PCR group was identified by sequencing the 16S rRNA gene and comparing to the type strain database using EzTaxon v 2.1 software (Tables 2 and S2). Most of the soil isolates (*i.e.*, 26) had sequences identical or almost identical (98–100% pairwise sequence similarity) to the species *Mycobacterium septicum*, 2 isolates to *Mycobacterium fortuitum* subsp. *acetamidolyticum*, 1 isolate to *Mycobacterium alvei*, 1 isolate to *Mycobacterium litorale* and 1 isolate to *Mycobacterium aubagnense*. The isolates from one BOX-PCR group were usually assigned to the same species, with the exception of group A, where isolates were attributed either to *M. septicum* or to *M. litorale*, group C where one isolate was attributed to *M. alvei* and another to *M. septicum*, and group I (3 isolates attributed to *M. septicum* and one to *M. fortuitum*). The set of clinical isolates was more diverse and comprised 17 species, of which *M. fortuitum* was the most prevalent (29%), followed by *M. neoaurum* (16%) (Table 3 and S3). As in the case of the soil isolates, different species sometimes occurred in the same BOX-PCR group, *e.g.*, *M. septicum* and *M. fortuitum* in group R, *M. goodie* and *M. neoaurum* in group V, and *M. abscessus* and *M. fortuitum* in group λ.

### Resistance to tetracycline

**Fig. 1:**
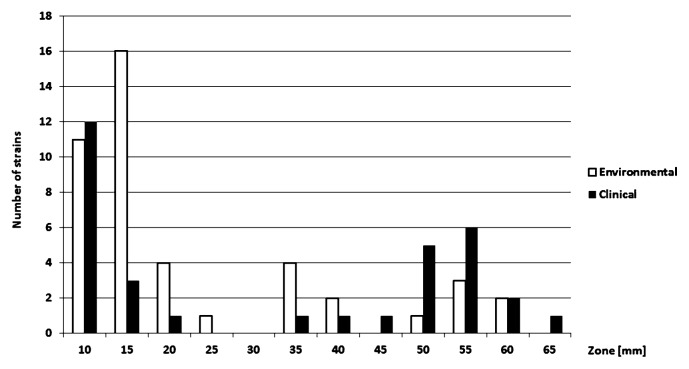
Tetracycline resistance in the clinical and the environmental isolates of rapidly growing mycobacteria. Bars represent the number of isolates with the corresponding inhibition zone size around 30 μg tetracycline disks.

The distribution of TET resistance was bimodal in both clinical and environmental strains (Fig. 1). Since standard breakpoints of the disc diffusion method are not available for RGM, we arbitrarily set them according to the zone size distributions. An isolate was considered to be resistant if the zone was up to 25 mm. Based on the arbitrary breakpoint, thirty-two environmental (70%) and sixteen clinical isolates (53%) were resistant to TET (Table 2 and 3). There was no significant difference in TET resistance (in terms of zone sizes, assessed with Wilcoxon rank sum test) between the environmental and clinical isolates. Tetracycline resistance was not a specific characteristic of individual BOX-PCR groups, *i.e.*, both resistant and sensitive isolates could be found within the same BOX-PCR group.

### Detection of tetracycline resistance genes

The two primer pairs designed in this study, *i.e.*, tetV-FW/tetV-RV [*tet*(V) detection], and tetKL-FW/tetKL-RV [simultaneous detection of *tet*(K) and *tet*(L)], were tested for their specificity in PCR using different tetracycline efflux pump genes as templates. Both primer pairs were specific, *i.e.*, they amplified only *tet*(V) in the case of tetV-FW/tetV-RV and *tet*(K) and *tet*(L) in the case of tetKL-FW/tetKL-RV (data not shown). The specificity of the tetV-FW/tetV-RV primer pair was further corroborated by sequencing the PCR products (see below).

All isolates were tested for the presence of *otr*(A), *otr*(B), *tet*(K)/*tet*(L), *tet*(M), *tet*(V) and *tap*. The gene *tet*(V) was detected in 32 of the total 44 environmental strains (73%) and in fourteen clinical isolates (37%) (Table 2 and 3). The environmental isolates harboring *tet*(V) were assigned to the species *M. septicum*, *M. litorale*, *M. alvei* and *M. fortuitum*. The clinical isolates with *tet*(V) belonged to the species *M. fortuitum*, *M. peregrinum*, *M. septicum*, *M. goodii*, *M. arupense* and *M. neoaurum*. Surprisingly, *tet*(V) was found also in isolates that were sensitive to tetracycline (even in those with zones over 50 mm) (Table 2 and 3). The gene *tap* was detected in 37 environmental isolates (84%) and in 28 clinical isolates (74%). The environmental isolates with *tap* belonged to the species *M. fortuitum*, *M. alvei*, *M. septicum*, and the clinical isolates to the species *M. fortuitum*, *M. novacastrense*, *M. peregrinum*, *M. septicum*, *M. goodi*, *M. neoaurum*, *M. rufum*, *M. obuense*, *M. frederiksbergense* and *M. mucogenicum*. Similarly to *tet*(V), *tap* was detected also in strains that were sensitive to tetracycline. In contrast, the genes *otr*(A), *otr*(B), *tet*(K)/*tet*(L) and *tet*(M) were not detected in any isolate, and eight clinical and seven environmental isolates were resistant to TET but possessed none of the tested genes.

### Analyses of *tet*(V) and *tap* sequences

The gene *tet*(V) was sequenced in all isolates (*i.e.*, 46) where we obtained positive signals from PCR. Sequences of isolates Site2-3C, Site2-5C and Site2-7C (all from the BOX-PCR group H) were not used for further analyses since they contained several ambiguous peaks that could not be resolved (although PCR and sequencing were attempted twice). It is possible that these isolates had two copies of *tet*(V) that differed slightly in their sequences.

**Fig. 2:**
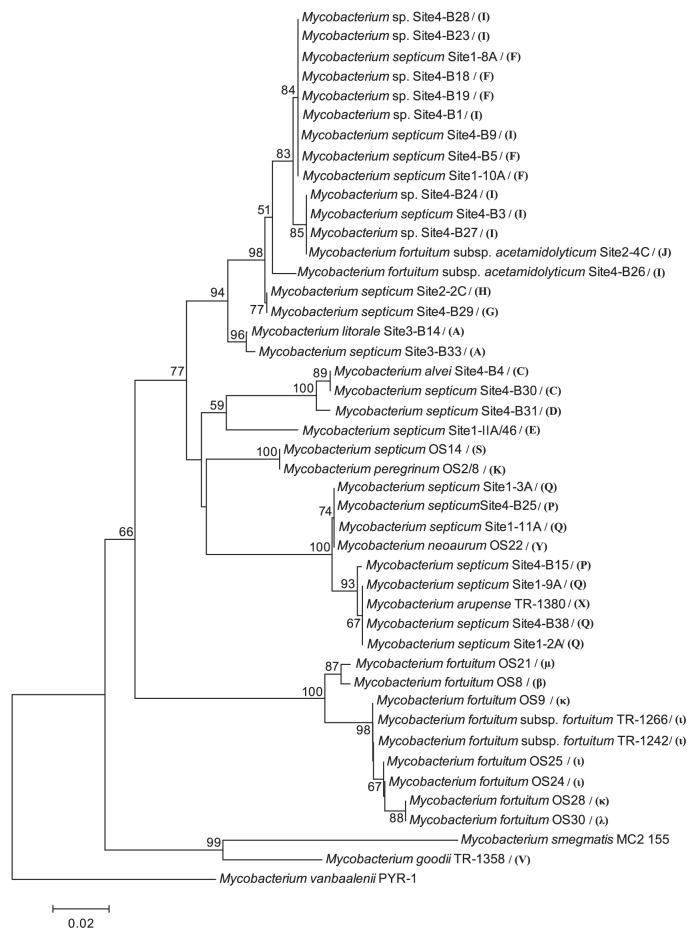
Phylogenetic tree based on the nucleotide sequences of *tet*(V), constructed by the neighbor-joining method using Kimura-2 parameter. Bootstrap values are indicated at the nodes as a percentage of 1,000 replications, if they were higher than 50%. Letters following the isolate names indicate the BOX-PCR groups the isolates belonged to.

**Fig. 3:**
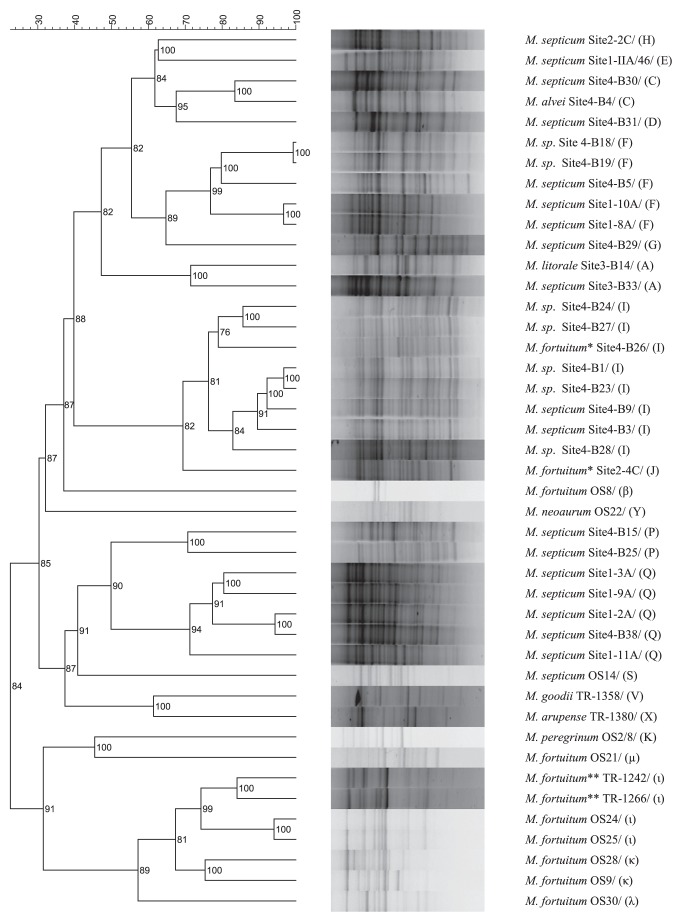
BOX-PCR profiles of 43 RGM isolates with sequenced *tet*(V). Left, UPGMA-clustering of the isolates based on the similarity matrix of their BOX-PCR profiles. Right, isolate names preceded by a letter indicating the BOX-PCR groups (based on the ≥70% similarity threshold). See Fig. S1 for BOX-PCR profiles of all isolates from this study.

The 43 analyzed partial sequences of the gene *tet*(V) [corresponding to positions 823–1,132 of *M. smegmatis* MC2-155 *tet*(V)] differed substantially among the isolates, with 17% differences between two most distant isolates (Fig. 2). The isolates shared 82–88% and 90–95% identity, respectively, of nucleic and inferred amino acid sequences with the published *tet*(V) sequence of *M. smegmatis* MC2-155. Since all the recovered sequences had amino acid identity above 80% with the published *tet*(V), they can be attributed to the *tet*(V) gene class (32). Interestingly, *M. neoarum* OS22 had a one-nucleotide deletion at position 915. The phylogeny of *tet*(V) (Fig. 2) shared overall similarity with the genome relatedness of the strains (Fig. 3), and the soil isolates usually clustered separately from clinical RGM. Indeed, there was a significant correlation (*r*=0.64, *P*=0.001) between the BOX-PCR profile- and *tet*(V) sequence-based distant matrices.

Fourteen PCR products of *tap* (corresponding to positions 764–1,080 of *tap M. fortuitum*) (4) were chosen for sequencing in order to verify the specificity of primers and check for *tap* gene diversity. Similarly to *tet*(V), differences in the partial sequences of *tap* between our isolates and the published sequence were found (data not shown). Out of the sequenced isolates, *M. fortuitum* subsp. *fortuitum* TR-1242 had the *tap* sequence most similar to the published sequence (99% identity), while the *M. rufum* TR-1359 *tap* sequence was the most dissimilar (only 79% identity). The environmental isolates had 82–89% sequence identity with the published *tap* sequence, and there was quite a high diversity of the sequences among the environmental isolates (up to 15% substitutions). The number of differences was lower at the amino acid level (96–100% amino acid identities).

## Discussion

In general, the distribution of TET resistance did not differ between the soil and the clinical RGM and the same TET-resistance genes were found in both groups. The gene *tet*(V) encoding a tetracycline efflux pump (19) was found in 73% soil isolates and 37% clinical isolates, including species where it has not been reported previously, *i.e.*, *M. septicum*, *M. litorale*, *M. alvei*, *M. peregrinum*, *M. goodii*, *M. arupense* and *M. neoaurum*. This study also showed the high diversity of *tet*(V) (up to 17% difference between two sequences), which is unusual among other *tet* gene classes. For example, the published sequences of *tet*(B), a horizontally transferred TET efflux pump with at least 20 reported host genera (40), did not differ from each other in more than 1% nucleotides (analysis not shown). This finding, together with the positive correlation between the BOX-PCR profiles and *tet*(V) phylogeny, suggest that *tet*(V) evolved together with the mycobacterial genomes rather than being acquired horizontally. These findings therefore support the hypothesis that *tet*(V) belongs to the mycobacterium intrinsic resistome; however, in certain cases, different RGM species (*e.g.*, *M. arupense* and *M. septicum*) shared the same partial *tet*(V) sequence, so the horizontal exchange of *tet*(V) among mycobacteria cannot be completely excluded. It is also conceivable that this gene underwent a long evolution within a certain group of RGM species from which it was horizontally transferred to other RGM species later. Comparative studies of the *tet*(V) gene and its sourroundings in a broader set of RGM species might be the next step to learn more about antibiotic resistance evolution in mycobacteria.

BLAST search revealed the presence of *tet*(V) homologues in the sequenced genomes of seven mycobacterial strains and in seven other actinomycetes; for example, there was a gene for H+ antiporter of *M. vanbaalenii* PYR-1 (86% identity), *Mycobacterium* sp. JLS (CP000580.1, 82% identity), *Mycobacterium* sp. KMS (CP000518.1, 82% identity) or major facilitator superfamily protein of *Geodermatophilus obscurus* DSM 43160 (CP001867.1, 73% identity). These genes may potentially code for drug resistance and therefore merit further attention. It is also possible that *tet*(V) is present in more mycobacterial species but has not been detected with PCR because of its high diversity and thus possible lack of complementarity with the primers used here.

The gene *tap*, which confers low-level resistance to tetracycline and certain aminoglycosides (4), seems to be very common among RGM. In our study, we found it in 84% environmental and 74% clinical isolates. Likewise, Esteban *et al.* (22) found *tap* in most (66%) of the clinical RGM tested. Besides the species in which *tap* has already been reported (22), it was detected also in *M. litorale*, *M. novacastrense*, *M. septicum*, *M. goodi*, *M. neoaurum*, *M. rufum*, *M. obuense*, *M. frederiksbergense* and *M. mucogenicum*. Based on the number of identities with the published *tap* sequence, it seems that we recovered more diverse *tap* sequences (83–99% identity) than Esteban *et al.* (22) (92–95% identity), using the same primers. Analysis of more sequences would be necessary to show whether there is a correlation between BOX-PCR profiles and *tap* phylogeny, as in the case of *tet*(V).

Surprisingly, *tet*(V) and/or *tap* were found also in the isolates that were highly susceptible to TET. Although our sequences of Tet(V) differed from that for which functionality was previously shown (19), there was no correlation between the *tet*(V) alleles and TET inhibition zone size; for example, two clinical *M. fortuitum* had the same sequence (at least in the part analyzed) but markedly differed in resistance. In general, sense mutations prevailed over the mutations changing the amino acid sequence, indicating that the function was rather to be maintained; however, *M. neoaurum* OS22 had one nucleotide deletion in the analyzed part of *tet*(V) that would change the reading frame, and this isolate was sensitive to tetracycline. It is possible that point deletions in the unanalyzed parts of the *tet*(V) sequence occurred also in other isolates. In addition, the observed discrepancy between genotype and phenotype may be due to mutations in distant regulation regions or to gene expression regulation; for example, Nash *et al.* (35) noticed that high clarithromycin resistance was inducible by overnight incubation of certain sensitive mycobacteria with a low concentration of the antibiotic. The discrepancy between the presence of a resistance gene and the resistance to TET in RGM or other bacteria was reported previously (5, 22). TET-resistant isolates in which *tet*(V) and *tap* were not detected could either differ in their *tet*(V) and *tap* sequences from the used primers or possess other TET resistance genes that were not tested. The search for horizontally transferred TET resistance genes so far described in RGM was unsuccessful, though it included the genes *tet*(M) and *tet*(L), which are commonly found in Gram-positive bacteria in manure (1, 3). The number of studies reporting horizontally transferred TET resistance genes in RGM is low (38, 41) and it could be that there are marked local differences in the distribution of these genes in RGM.

The majority of the soil as well as clinical isolates from this study belonged to the *Mycobacterium fortuitum* group. Most soil isolates had the highest 16S rRNA sequence similarity to the species *M. peregrinum*, *M. septicum* or *M. fortuitum*, which all include potential human pathogens (29, 48). Some of the isolates close to *M. septicum* indeed grew at 37°C (data not shown), indicating they may be able to colonize the human body; however, the soil and clinical isolates of *M. septicum* from this study were genetically different, as shown by analyses of their BOX-PCR profiles (and the same applies also to *M. fortuitum* isolates). The species in the *M. fortuitum* group are frequently recovered from soil (25), including agricultural soils (20, 53), as well as from clinical samples (24, 50), but so far a direct relationship between a clinical manifestation and exposure to soil has not been clearly shown in this group. The group *M. chelonae*-*M. abscessus* was reported as being even more frequent than the *M. fortuitum* group in clinical samples (24, 50), but not in our case. The discrepancy between BOX-PCR groups and the assignment of isolates to species (*e.g.*, BOX-PCR groups A, C or I) can be because the BOX-PCR groups were defined quite broadly (70% similarity) (12), but it can also indicate the need for more comprehensive taxonomical evaluation of RGM, as already shown in the case of streptomycetes (31).

In this study, we tested resistance to tetracycline and not to clinically used doxycycline, which was relevant in the context of soil since tetracycline residues are found in soil and manure (10). Ultimately, the zone sizes with tetracycline and doxycycline disks were correlated (tested with 32 isolates, Pearson correlation 0.88, *P*<0.01, data not shown). It was previously shown with the disk dilution method that RGM susceptible to tetracycline were also susceptible to doxycline, although the MIC values were 1–2 dilutions lower for doxycycline (52). Interestingly, there was no difference in the zone size distribution between clinical and environmental RGM and more than half of the isolates from both groups were resistant to tetracycline. The bimodal distribution of resistance to tetracycline antibiotics was reported previously (44, 52). Both resistant and sensitive phenotypes occurred within one species (*e.g.*, within *M. fortuitum* or within *M. septicum*), and even within one BOX-PCR group (Table 2 and 3). We thus did not observe any consistency between BOX-PCR fingerprints and antibiotic resistance phenotypes, in contrast to Davelos Baines *et al.* (13), who found significant correlations between antibiotic phenotypes and BOX-PCR fingerprints in soil streptomycetes. This could be because the selection pressure for antibiotic resistance in soil acts on a much smaller scale than we sampled (13) and because antibiotic resistance in individual mycobacterial isolates can be affected by point mutations that would not significantly affect their BOX-PCR profiles. The variable resistance to tetracycline antibiotics within the *M. fortuitum* group is consistent with previous studies, usually reporting 40–60% sensitive isolates (24, 44, 52).

## Conclusion

In conclusion, the studied soil and the clinical RGM from the Czech Republic did not differ in the distribution of TET resistance and occurrence of TET resistance genes, most possessing efflux-pump encoding genes *tet*(V) and/or *tap*. This study shows for the first time the presence of *tet*(V) and *tap* in soil mycobacteria. The correlation between the *tet*(V) phylogeny and isolate genomic profiles indicates that *tet*(V) belongs to the mycobacterial intrinsic resistom. The intrinsic efflux pumps may therefore play an important role in the antibiotic resistance of RGM, as in the case of *M. tuberculosis (*18, 33). The simple presence of efflux-pump encoding genes, however, did not always match TET resistance. Further research should therefore be performed on gene diversity, distribution and expression in order to better understand the mycobacterial intrinsic resistom.

## Supplementary Material



## Figures and Tables

**Table 1:** Primers and PCR conditions used for tetracycline resistance gene amplification

Gene	Primers	Primer sequences 5′-3′ (Reference)	PCR cycles	Amplicon (bp)	Positive control
*otr*(A)	otr(A) (F) otr(A) (R)	GAACACGTACTGACCGAGAAG CAGAAGTAGTTGTGCGTCCG (37)	5 min/95°C; 35×(1 min/94°C, 30 s/60°C, 30 s/72°C); 5 min/72°C	778	*Streptomyces rimosus* subsp. *rimosus* DSMZ 40260 (ATTC 10970)
*otr*(B)	otr(B) (F) otr(B) (R)	CCGACATCTACGGGCGCAAGC GGTGATGACGGTCTGGGACAG (37)	5 min/95°C; 35×(1 min/94°C, 1 min/68°C, 1 min/72°C); 7 min/72°C	947	*Streptomyces rimosus* subsp. *rimosus* DSMZ 40260 (ATTC 10970)
*tap*	Tap1 Tap2	GTCGCGTTCCCGTGGCTGGT CGATACCGGGGCCGACGATG (22)	10 min/94°C; 35×(1 min/94°C, 30 s/68°C, 30 s/72°C); 3 min/72°C	400	*Mycobacterium fortuitum* TR-1242 (this study)
*tet*(K) and *tet*(L)	tetKL-FW tetKL-RV	TTACCTGATATTGCAA GACCAATGAATATAAT (this study)	5 min/95°C; 35×(30 s/94°C, 30 s/40°C, 30 s/72°C); 3 min/72°C	397	*Staphylococcus haemolyticus* CB-N (*tet*(K); this study) and *Staphylococcus aureus* pSTS9-like (*tet*(L); 1)
*tet*(M)	TetM-FW TetM-RV	ACAGAAAGCTTATTATATAAC TGGCGTGTCTATGATGTTCAC (6)	4 min/94°C; 35×(20 s/94°C, 30 s/52.3°C, 1 min/72°C); 7 min/68°C	171	Plasmid pAT101 that carries *tet*(M) gene from *Streptococcus* transposon Tn1545 (34)
*tet*(V)	tetV-FW tetV-RV	GCCTACGGTTTCATCCTGGC CGAGACCACCTTCGACAGCG (this study)	7 min/95°C; 35×(1 min/94°C, 15 s/65°C, 30 s/72°C); 5 min/ 72°C	351	*Mycobacterium* sp. Site2-2C (this study)

**Table 2:** Tetracycline resistance and presence of resistance genes in the environmental isolates

Isolate	BOX group	Identification^a^	TET resistance (zone in mm)^b^	Presence of gene^c^	

				*tap*	*tet*(V)
Site3-B14	A	*M. litorale* (99.46%)	**12**	+	+
Site3-B33	A	*M. septicum* (100%)	35	+	+
Site4-B4	C	*M. alvei* (98.99%)	**9**	+	+
Site4-B30	C	*M. septicum* (100%)	**12**	+	+
Site4-B31	D	*M. septicum* (99.06%)	**11**	+	+
Site1-IIA/46	E	*M. septicum* (99.84%)	**12**	+	+
Site4-B5	F	*M. septicum* (99.58%)	**20**	+	+
Site4-B18	F	ND	**25**	+	+
Site4-B19	F	ND	**18**	+	+
Site1-8A	F	*M. septicum* (99.93%)	**14**	+	+
Site1-10A	F	*M. septicum* (100%)	**13**	+	+
Site4-B2	G	*M. septicum* (100%)	**11**	+	−
Site4-B7	G	ND	**15**	+	−
Site4-B8	G	ND	**9**	−	−
Site4-B29	G	*M. septicum* (99.77%)	**11**	+	+
Site2-2C	H	*M. septicum* (100%)	**14**	+	+
Site2-3C	H	*M. septicum* (100%)	**14**	+	+
Site2-5C	H	*M. septicum* (99.78%)	**17**	+	+
Site2-7C	H	*M. septicum* (99.76%)	**10**	+	+
Site4-B1	I	*M. septicum* (99.55%)	**11**	+	+
Site4-B3	I	*M. septicum* (98.93%)	37	+	+
Site4-B9	I	*M. septicum* (99.25%)	**11**	+	+
Site4-B23	I	ND	**10**	+	+
Site4-B24	I	ND	39	+	+
Site4-B26	I	*M. fortuitum* subsp. *acetamidolyticum* (99.46%)	35	+	+
Site4-B27	I	ND	35	+	+
Site4-B28	I	ND	**10**	+	+
Site2-4C	J	*M. fortuitum* subsp. *acetamidolyticum* (99.42%)	31	+	+
Site4-B6	M	*M. septicum* (99.17%)	**10**	−	−
Site4-B16	M	ND	**9**	−	−
Site4-B17	M	ND	**8**	−	−
Site4-B21	M	ND	**9**	−	−
Site4-B39	M	ND	**20**	−	−
Site4-B15	P	*M. septicum* (99.71%)	50	+	+
Site4-B25	P	*M. septicum* (99.56%)	55	+	+
Site4-B38	Q	*M. septicum* (100%)	**14**	+	+
Site1-2A	Q	*M. septicum* (99.71%)	53	+	+
Site1-3A	Q	*M. septicum* (99.71%)	56	+	+
Site1-9A	Q	*M. septicum* (99.71%)	56	+	+
Site1-11A	Q	*M. septicum* (99.71%)	52	+	+
Site3-B10	U	*M. septicum* (100%)	**15**	+	−
Site3-B34	U	ND	**10**	+	−
Site4-B36	U	*M. septicum* (99.67%)	**10**	+	−
Site2-IIIC/14	δ	*M. aubagnense* (99.04%)	**12**	−	−

a In parentheses, % pairwise sequence similarity with the closest type strain on EzTaxon is shown. ND, not done.

b Resistance in bold.

c The genes *otr*(A), *otr*(B), *tet*(K)(L) and *tet*(M) were detected in none of the isolates.

**Table 3:** Tetracycline resistance and presence of resistance genes in the clinical isolates

Isolate	BOX group	Identification	TET resistance (zone in mm)	Presence of gene^b^	

				*tap*	*tet*(V)
OS6	B	*M. novacastrense* (99.56%)	60*	+	−
OS2/8	K	*M. peregrinum*^a^	**6.5**	+	+
TR-1378	L	*M. fortuitum*^a^	**17**	+	−
OS2/2	N	*M. arupense* (100%)	6.5*	−	−
TR-1536	O	*M. franklinii* (100%)	**6.5**	−	−
OS19	R	*M. septicum* (100%)	**6.5**	+	−
OS2/7	R	*M. fortuitum*^a^	**11**	+	−
OS14	S	*M. septicum* (100%)	**11**	+	+
OS16	T	*M. septicum* (100%)	**6.5**	+	−
TR-1358	V	*M. goodii* (99.77%)	41	+	+
OS2	V	*M. neoaurum* (99.37%)	58	+	−
TR-1344	W	*M. llatzerense* (98.40%)	**11**	−	−
TR-1380	X	*M. arupense* (98.95%)	55	−	+
OS22	Y	*M. neoaurum* (100%)	48	+	+
OS29	Y	*M. neoaurum* (100%)	54	+	−
OS2/1	Z	*M. nonchromogenicum* (98.91%)	6.5*	−	−
TR-1294	α	*M. neoaurum* (99.76%)	47	+	−
OS3	α	*M. neoaurum* (100%)	48	+	−
OS8	β	*M. fortuitum*^a^	**9**	+	+
OS26	γ	*M. neoaurum* (100%)	57	+	−
OS27	ɛ	*M. obuense* (99.78%)	48	+	−
OS4	ζ	*M. rufum* (100%)	52	+	−
TR-1359	η	*M. rufum* (99.76%)	61	+	−
OS13	θ	*M. abscessus*^a^	**9**	−	−
TR-1242	ι	*M. fortuitum* subsp. *fortuitum* (99.88%)	52	+	+
TR-1266	ι	*M. fortuitum* subsp. *fortuitum* (100%)	**6.5**	+	+
OS24	ι	*M. fortuitum*^a^	**6.5**	+	+
OS25	ι	*M. fortuitum*^a^	**6.5**	+	+
OS9	κ	*M. fortuitum*^a^	**6.5**	+	+
OS28	κ	*M. fortuitum*^a^	55	+	+
OS10	λ	*M. abscessus*^a^	**6.5**	−	−
OS30	λ	*M. fortuitum*^a^	**6.5**	+	+
OS21	μ	*M. fortuitum*^a^	31	+	+
OS18	ν	*M. hiberniae* (99.78%)	6.5*	−	−
OS2/4	ν	*M. hiberniae* (99.78%)	6.5*	−	−
OS11	ξ	*M. mucogenicum*^a^	49	+	−
OS7	π	*M. frederiksbergense* (99.56%)	55	+	−
OS1	ρ	*M. smegmatis*^a^	38	−	−

a Identified with the GenoType Mycobacterium CM (Common Mycobacteria) Test based on DNA Strip technology (Hain Lifescience)

b The genes *otr*(A), *otr*(B), *tet*(K)(L) and *tet*(M) were detected in none of the isolates.

* Measured on Šula’s medium (43).
