# Supplementary material for: Tetracycline Resistance and Presence of Tetracycline Resistance Determinants tet(V) and tap in Rapidly Growing Mycobacteria from Agricultural Soils and Clinical Isolates
Source: Microbes Environ. 2012 May 17;27(4):413–22. doi: 10.1264/jsme2.ME12028 (PMC4103549; doi:10.1264/jsme2.ME12028)
Supplement: Supplementary file 1 [file 27_413_s1.pdf]

Table S1. Description of sampled sites and soil characteristics. The values represent average ± standard deviation, n = 6.

Site	Farm	Description	pH[a] ($CaCl_2$)	C organic [a] (g kg^{-1})	N total [a] (g kg^{-1})	P available [a] (mg kg^{-1})	Microbial biomass[b] (nmol PLFA g^{-1})
1	Farm 1	Permanent grassland periodically manured for past 30 years with pig slurry from antibiotic (chlortetracycline, doxycycline, amoxyciline, fluorophenicol) treated pigs.	5.7±0.1	89±10.2	2.4±0.2	89±8.6	14.8±1.8
2	Farm 2	Permanent grassland, not manured for past 20 years.	5.9±0.0	63±8.9	1.4±0.0	63.0±3.5	10.6±0.7
3	Farm 3	Pasture highly impacted by cattle overwintering (excrement deposits) in an organic farm without antibiotic administration.	7.4±0.3	155.0±16.5	6.5±0.2	671.6±58.2	119.0±20.1
4	Farm 3	Control pasture with low impact of cattle in an organic farm without antibiotic administration.	4.9±0.1	59.4±8.5	1.8±0.1	8.78±3.3	38.3±23.1

[a]The physical and chemical characteristics of the sampled soils were measured in the accredited laboratory AGRO-LA, spol. s r.o. (Jindrichuv Hradec, Czech Republic). The concentration of available phosphorus (P available) was measured according to Mehlich III (1984), total nitrogen (N total) was measured using the Kjeldahl mineralization method (Kirk, 1950), and the amount of organic carbon (C organic) was measured using the incineration method (Jackson, 1958).

[b]The microbial biomass was measured as the total content of phospholipid fatty acids (Elhottová *et al*., 2009).

References:

Elhottová, D., V. Krištůfek, S. Malý, and J. Frouz. 2009. Rhizosphere effect of colonizer plant species on the development of soil microbial community during primary succession on postmining sites. Comm Soil Sci Plant Anal 40: 758–770).

Jackson, M.L. 1958. Soil chemical analysis. Prentice–Hall, Englewood Cliffs, NJ.

Kirk, P.L. 1950. Kjeldahl method for total nitrogen. Anal. Chem. 22:354-358.

Mehlich, A. 1984. Mehlich-3 soil test extractant: a modification of Mehlich-2 extractant. Commun. Soil Sci. Plan. 15:1409-1416.

Table S2. List of the environmental isolates

Isolate	Identification[a]	Year of isolation	Farm	Site characteristic	Pretreatment method[a]	Medium[b]
Site1-2A	*M. septicum* (99.711%)	2009	1	Manured with pig slurry	NaOH/malachite green/cycloheximide	M2
Site1-3A	*M. septicum* (99.711%)	2009	1	Manured with pig slurry	NaOH/malachite green/cycloheximide	Ogawa
Site1-8A	*M. septicum* (99.928%)	2009	1	Manured with pig slurry	NaOH/malachite green/cycloheximide	M2
Site1-9A	*M. septicum* (99.708%)	2009	1	Manured with pig slurry	NaOH/malachite green/cycloheximide	M2
Site1-10A	*M. septicum* (100%)	2009	1	Manured with pig slurry	NaOH/malachite green/cycloheximide	MYM
Site1-11A	*M. septicum* (99.711%)	2009	1	Manured with pig slurry	NaOH/malachite green/cycloheximide	M2
Site1-IIA/46	*M. septicum* (99.838%)	2007	1	Manured with pig slurry	Soil preincubaction with 1 mg TET/g dry soil	TSA+TET
Site2-2C	*M. septicum* (100%)	2009	2	Non-manured	NaOH/malachite green/cycloheximide	M2
Site2-3C	*M. septicum* (100%)	2009	2	Non-manured	NaOH/malachite green/cycloheximide	Ogawa
Site2-4C	*M. fortuitum subsp. acetamidolyticum*	2009	2	Non-manured	NaOH/malachite green/cycloheximide	MYM

	(99.421%)					
Site2-5C	*M. septicum* (99.783%)	2009	2	Non-manured	NaOH/malachite green/cycloheximide	M2
Site2-7C	*M. septicum* (99.760%)	2009	2	Non-manured	NaOH/malachite green/cycloheximide	MYM
Site2-IIIC/14	*M. aubagnense* (99.043%)	2007	2	Non-manured	Soil preincubaction with 0,1 mg TET/g dry soil	TSA+TET
Site3-B10	*M. septicum* (100%)	2010	3	Strong cattle impact	Olive oil/SDS	TSA
Site3-B14	*M. litorale* (99.46%)	2010	3	Strong cattle impact	NaOH/malachite green/cycloheximide	M2
Site3-B33	*M. septicum* (100%)	2010	3	Strong cattle impact	NaOH/malachite green/cycloheximide	HV
Site3-B34	ND	2010	3	Strong cattle impact	NaOH/malachite green/cycloheximide	HV
Site4-B1	*M. septicum* (99.551%)	2010	3	Low cattle impact	Olive oil/SDS	TSA
Site4-B2	*M. septicum* (100%)	2010	3	Low cattle impact	Olive oil/SDS	HV
Site4-B3	*M. septicum* (98.932%)	2010	3	Low cattle impact	Olive oil/SDS	HV
Site4-B4	*M. alvei* (98.988%)	2010	3	Low cattle impact	Olive oil/SDS	M2
Site4-B5	*M. septicum* (99.578%)	2010	3	Low cattle impact	Olive oil/SDS	M2
Site4-B6	*M. septicum* (99.169%)	2010	3	Low cattle impact	Olive oil/SDS	M2
Site4-B7	ND	2010	3	Low cattle impact	Olive oil/SDS	M2
Site4-B8	ND	2010	3	Low cattle impact	Olive oil/SDS	M2
Site4-B9	*M. septicum* (99.251%)	2010	3	Low cattle impact	Olive oil/SDS	M2

Site4-B15	*M. septicum* (99.713%)	2010	3	Low cattle impact	NaOH/malachite green/cycloheximide	M2
Site4-B16	ND	2010	3	Low cattle impact	NaOH/malachite green/cycloheximide	M2
Site4-B17	ND	2010	3	Low cattle impact	NaOH/malachite green/cycloheximide	M2
Site4-B18	ND	2010	3	Low cattle impact	NaOH/malachite green/cycloheximide	M2
Site4-B19	ND	2010	3	Low cattle impact	NaOH/malachite green/cycloheximide	M2
Site4-B21	ND	2010	3	Low cattle impact	NaOH/malachite green/cycloheximide	M2
Site4-B23	ND	2010	3	Low cattle impact	NaOH/malachite green/cycloheximide	HV
Site4-B24	ND	2010	3	Low cattle impact	NaOH/malachite green/cycloheximide	HV
Site4-B25	*M. septicum* (99.561%)	2010	3	Low cattle impact	NaOH/malachite green/cycloheximide	HV
Site4-B26	*M. fortuitum subsp. acetamidolyticum* (99.459%)	2010	3	Low cattle impact	NaOH/malachite green/cycloheximide	HV
Site4-B27	ND	2010	3	Low cattle impact	NaOH/malachite green/cycloheximide	HV
Site4-B28	ND	2010	3	Low cattle impact	NaOH/malachite green/cycloheximide	TSA
Site4-B29	*M. septicum* (99.764%)	2010	3	Low cattle impact	NaOH/malachite green/cycloheximide	TSA
Site4-B30	*M. septicum* (100%)	2010	3	Low cattle impact	NaOH/malachite green/cycloheximide	TSA
Site4-B31	*M. septicum* (99.056%)	2010	3	Low cattle impact	NaOH/malachite green/cycloheximide	TSA
Site4-B36	*M. septicum* (99.671%)	2010	3	Low cattle impact	NaOH/malachite green/cycloheximide	TSA

Site4-B38	*M. septicum* (100%)	2010	3	Low cattle impact	NaOH/malachite green/cycloheximide	TSA
Site4-B39	ND	2010	3	Low cattle impact	NaOH/malachite green/cycloheximide	TSA

[a] Pairwise 16S rDNA sequence similarity % with the closest type species on EzTaxon database. ND, not done.

[b]TSA, Tryptic-Soy agar; TSA+TET, TSA supplemented with 25 mg L^{-1} chlortetracycline; M2, M2 medium (Shirling and Gottlieb, 1966); Ogawa, Ogawa egg agar (Tsukamura *et al*., 1986); HV, Humic acid-Vitamin agar (Hayakawa and Nonomura, 1987); MYM, MYM medium (source: The Czech National Collection of Type Cultures; formula for 1 liter: yeast extract 2 g, proteose peptone No.3 2 g, casitone 2 g, $Na_2HPO_4.12H_2O$ 2.5 g, KH_2PO_4 1 g, sodium citrate 1.5 g, $MgSO_4.7H_2O$ 0.6 g, Tween 80 0.5 g, glycerol 50 mL, agar 20 g, pH 7.0).

References:

Hayakawa, M., and H. Nonomura. 1987. Humic acid-vitamin agar, a new medium for the selective isolation of soil actinomycetes. J. Ferment. Technol. 65:501–509.

Shirling, E.B., and D. Gottlieb. 1966. Methods for characterization of streptomycetes. Int. J. Syst. Bacteriol. 16:313–340.

Tsukamura, M., I. Yano, and T. Imaeda. 1986. *Mycobacterium moriokaense* sp. nov., a rapidly growing, non photochromogenic mycobacterium. Int. J. Syst. Bacteriol. 36:333–338.

Table S3. List of the clinical isolates

Isolate	Identification	Method of identification[a]	Year of isolation	Hospital	Site	Pretreatment method	Medium[b]
TR-1242	*M. fortuitum* subsp. *fortuitum*	Sequencing (99.880%)	2007	Duchcov	Abcess	-	Blood
TR-1266	*M. fortuitum* subsp. *fortuitum*	Sequencing (100%)	2009	Pribram	Sputum	Sodium lauryl sulfate	LW
TR-1294	*M. neoaurum*	Sequencing (99.758%)	2008	Trutnov	Sputum	NaOH	Ogawa
TR-1344	*M. llatzerense*	Sequencing (98.397%)	2008	Praha Bulovka	Hemoculture	-	Blood
TR-1358	*M. goodii*	Sequencing (99.768%)	2008	Nachod	Sputum	Sodium lauryl sulfate	LW
TR-1359	*M. rufum*	Sequencing (99.76%)	2008	Pribram	Sputum	Sodium lauryl sulfate	LW
TR-1378	*M. fortuitum*	DNA®Strip technology	2008	Trutnov	Sputum	NaOH	Ogawa

TR-1380	*M. arupense*	Sequencing (98.947%)	2009	Trutnov	Sputum	NaOH	Ogawa
TR-1536	*M. franklinii*	Sequencing (100%)	2010	Kromeriz	Corneal ulcer	-	Blood
OS1	*M. smegmatis*	DNA®Strip technology	2010	Karvina	Sputum	NaOH	LW
OS2	*M. neoaurum*	Sequencing (99.374%)	2008	Novy Jicin	Sputum	NaOH	LW
OS3	*M. neoaurum*	Sequencing (100%)	2009	Frydek-Mistek	Sputum	NaOH	LW
OS4	*M. rufum*	Sequencing (100%)	2009	Bruntal	Sputum	NaOH	LW
OS6	*M. novacastrense*	Sequencing (99.559%)	2010	Frydek-Mistek	Sputum	NaOH	LW
OS7	*M. frederiksbergense*	Sequencing (99.555%)	2006	Jablunkov	Sputum	NaOH	LW
OS8	*M. fortuitum*	DNA®Strip technology	2010	Ostrava	Sputum	NaOH	LW

OS9	*M. fortuitum*	DNA®Strip technology	2010	Bohumin	Sputum	NaOH	LW
OS10	*M. abscessus*	DNA®Strip technology	2010	Havirov	Sputum	NaOH	LW
OS11	*M. mucogenicum*	DNA®Strip technology	2008	Ostrava	Sputum	NaOH	LW
OS13	*M. abscessus*	DNA®Strip technology	2009	Novy Jicin	Sputum	NaOH	LW
OS14	*M. septicum*	Sequencing (100%)	2010	Bruntal	Sputum	NaOH	LW
OS16	*M. septicum*	Sequencing (100%)	2009	Jevicko	Sputum	NaOH	LW
OS18	*M. hiberniae*	Sequencing (99.784%)	2011	Novy Jicin	Urine	NaOH	LW
OS19	*M. septicum*	Sequencing (100%)	2006	Krnov	Sputum	NaOH	LW
OS21	*M. fortuitum*	DNA®Strip technology	2008	Krnov	Sputum	NaOH	LW
OS22	*M. neoaurum*	Sequencing	2008	Ostrava	Sputum	NaOH	LW

		(100%)					
OS24	*M. fortuitum*	DNA®Strip technology	2008	Frenstat pod Radhostem	Sputum	NaOH	LW
OS25	*M. fortuitum*	DNA®Strip technology	2008	Frenstat pod Radhostem	Sputum	NaOH	LW
OS26	*M. neoaurum*	Sequencing (100%)	2009	Bruntal	Sputum	NaOH	LW
OS27	*M. obuense*	Sequencing (99.781%)	2009	Bruntal	Urine	NaOH	LW
OS28	*M. fortuitum*	DNA®Strip technology	2008	Ostrava	Sputum	NaOH	LW
OS29	*M. neoaurum*	Sequencing (100%)	2008	Ostrava	Urine	NaOH	LW
OS30	*M. fortuitum*	DNA®Strip technology	2008	Frydek-Mistek	Sputum	NaOH	LW
OS2/1	*M. nonchromogenicum*	Sequencing (98.913%)	2011	Frydek-Mistek	Sputum	NaOH	LW
OS2/2	*M. arupense*	Sequencing	2011	Krnov	Sputum	NaOH	LW

		(100%)					
OS2/4	*M. hiberniae*	Sequencing<br><br>(99.784%)	2011	Novy Jicin	Urine	NaOH	LW
OS2/7	*M. fortuitum*	DNA®Strip technology	2011	Zabreh na Morave	Sputum	NaOH	LW
OS2/8	*M. peregrinum*	DNA®Strip technology	2011	Ostrava	Sputum	NaOH	LW

[a]Sequencing: % show pairwise sequence similarity with closest type species on EzTaxon database. DNA®Strip technology: Identified with the Genotype Mycobacterium CM (Common Mycobacteria) Test based on DNA®Strip technology (Hain Lifescience, Nehren, Germany)

[b]Blood, blood agar; LW, Löwenstein–Jensen agar; Ogawa, Ogawa egg agar.

Table S4. Numbers of *Mycobacterium* isolates from soil obtained with different methods

Site and farm	Number of isolates per method and medium[a]			Total isolates
	Decontamination	Olive oil	Without pretreatment	
Site 1<br>(Farm 1)	M2 – 4<br>Ogawa – 1<br>MYM – 1		TSA with TET – 1	7
Site 2<br>(Farm 2)	M2 – 1<br>Ogawa – 1<br>MYM – 3		TSA with TET – 1	6
Site 3<br>(Farm 3)	M2 – 1<br>HV – 2<br>TSA – 0	M2 – 0<br>HV – 0<br>TSA – 1		4
Site 4<br>(Farm 3)	M2 – 6<br>HV – 5<br>TSA – 7	M2 – 6<br>HV – 2<br>TSA – 1		27
Total isolates	32	10	2	**44**

[a] TSA, Tryptic-Soy agar; TSA with TET, TSA supplemented with 25 mg L^{-1} chlortetracycline; M2, M2 medium (Shirling and Gottlieb, 1966); Ogawa, Ogawa egg agar (Tsukamura *et al*., 1986); HV, Humic acid-Vitamin agar (Hayakawa and Nonomura, 1987); MYM, MYM medium (source: The Czech National Collection of Type Cultures; formula for 1 liter: yeast extract 2 g, proteose peptone No.3 2 g, casitone 2 g, $Na_2HPO_4.12H_2O$ 2.5 g, KH_2PO_4 1 g, sodium citrate 1.5 g, $MgSO_4.7H_2O$ 0.6 g, Tween 80 0.5 g, glycerol 50 mL, agar 20 g, pH 7.0).

References:

Hayakawa, M., and H. Nonomura. 1987. Humic acid-vitamin agar, a new medium for the selective isolation of soil actinomycetes. J. Ferment. Technol. 65:501–509.

Shirling, E.B., and D. Gottlieb. 1966. Methods for characterization of streptomycetes. Int. J. Syst. Bacteriol. 16:313–340.

Tsukamura, M., I. Yano, and T. Imaeda. 1986. *Mycobacterium moriokaense* sp. nov., a rapidly growing, non photochromogenic mycobacterium. Int. J. Syst. Bacteriol. 36:333–338.

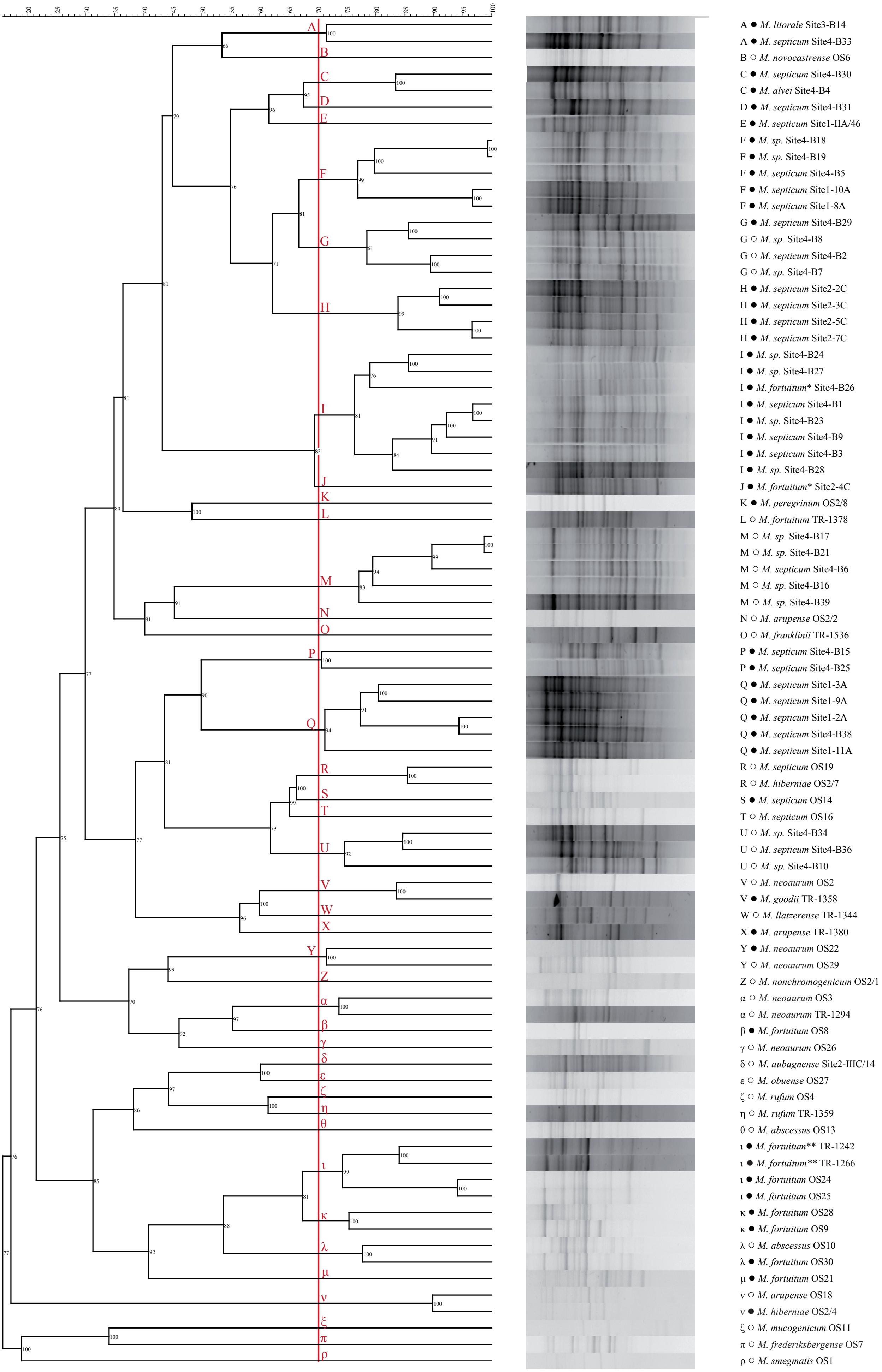

Fig. S1. BOX-PCR profiles from 82 studied *Mycobacterium* isolates. Left, UPGMA-clustering of the isolates based on the similarity matrix of their BOX-PCR profiles. The vertical line separates the branchings with ≥ 70% similarity (Pearson's r ≥ 0.7) between isolates. BOX-PCR groups are listed in greek alphabet. Right, isolate names preceded by a letter indicating the BOX-PCR groups (based on the ≥ 70% similarity threshold) and a circle indicating presence (full) or absence (empty) of the *tet(V)* gene. Most right, isolate identification based on 16S rRNA analysis (asterisk, *Mycobacterium fortuitum subsp. acetamidolyticum*; double asterisk, *Mycobacterium fortuitum subsp. fortuitum*).
